# Prevalence of common mental health disorders and associated factors among adolescents and young people living with HIV in Lusaka, Zambia

**DOI:** 10.1371/journal.pmen.0000710

**Published:** 2026-10-12

**Authors:** Kwame Shanaube, Suwilanji Simwanza, Buumba Tapisha, Dennis Ngosa, Lucheka Sigande, Isaac Mshanga, Choongo Mulungu, O’jay Mwenya, Jane Grace Mwamba, Lihlizulu Thabo Moyo, Charles Moyo, Temwani Mbambara, Gabriel Lungu, Madalitso Mbewe, Given Moonga, Brian Maila

**Affiliations:** 1 Zambart, Lusaka, Zambia; 2 CIDRZ, Analysis Unit, Lusaka, Zambia; 3 Ministry of Health, Clinical Care, Lusaka, Zambia; 4 University of Lusaka, Lusaka, Zambia; 5 Serenity Harm Reduction Programme Zambia, Lusaka, Zambia; 6 Ministry of Health, Lusaka, Zambia; 7 Lifeline Childline Zambia, Lusaka, Zambia; 8 Ministry of Health, Clinical Care and NCD Unit, Lusaka, Zambia; 9 Seed Global Health, Programs, Lusaka, Zambia; 10 Department of Epidemiology and Biostatistics, School of Public Health, University of Zambia, Lusaka, Zambia; 11 School of Medicine, University of Zambia, Lusaka, Zambia; PLOS: Public Library of Science, UNITED KINGDOM OF GREAT BRITAIN AND NORTHERN IRELAND

## Abstract

Mental health disorders (MHDs) are prevalent among adolescents and young people living with HIV (AYPLHIV) and can adversely affect wellbeing, antiretroviral therapy adherence and engagement in care. We aimed to determine the prevalence of common MHDs and associated factors among AYPLHIV receiving routine HIV care in Lusaka, Zambia. We conducted a cross-sectional survey embedded within the AdoMinds mixed-methods implementation study. The study was conducted from September 2023 to May 2024 at Chipata Level 1 Hospital, Lusaka. All AYP aged 15–24 years enrolled in ART care during the study period were invited to participate. A trained research assistant administered a tablet-based questionnaire including validated screening tools for depression, anxiety, post-traumatic stress disorder and substance use. Logistic regression was used to determine factors associated with MHDs. We enrolled 416 AYPLHIV (71.4% of 583 registered in care); 268 (64.4%) were female and the median age was 20 years (IQR: 18, 23). Overall 71.4% (297/416) screened positive for at least one MHD. Depression was most prevalent at 67.1% (279/416), followed by anxiety at 35.3% (147/416). Having at least one MHD was associated with feeling stigmatised because of HIV (AOR = 1.99, 95% CI: 1.10-3.63, p = 0.024). There was borderline evidence that a history of smoking was associated with higher odds of having at least one MHD (AOR = 4.24, 95% CI: 0.98-18.30, p = 0.053). Compared with participants with both parents alive, having lost one parent (AOR = 0.46, 95% CI: 0.21-0.97, p = 0.042) or both parents (AOR = 0.38, 95% CI: 0.18-0.80, p = 0.010) was associated with lower odds of having an MHD. Common MHDs were highly prevalent among AYPLHIV receiving routine HIV care, with HIV-related stigma associated with higher odds of MHDs. Integrating mental health screening and referral pathways into adolescent HIV services may support earlier identification and care.

## 1. Introduction

Mental health disorders (MHDs) are an important contributor to morbidity among adolescents and young people (AYP), with onset commonly occurring during adolescence and early adulthood [1,2]. Common MHDs, including depression, anxiety, post-traumatic stress disorder (PTSD) and substance-use disorders, can affect schooling, relationships, risk behaviours, treatment engagement and long-term wellbeing [3–6]. For adolescents and young people living with HIV (AYPLHIV), these effects are particularly important because MHDs may also compromise antiretroviral therapy (ART) adherence, clinic attendance and HIV treatment outcomes [7,8].

In Zambia, the burden of MHDs in the general population is estimated to be between 20% and 30% [9,10], while studies among AYP have linked mental distress with adverse childhood experiences, food insecurity, stigma, violence, substance use and HIV-related vulnerability [11–14]. Shortages of trained mental health personnel and limited infrastructure continue to affect the provision of mental health services in Zambia, contributing to a large treatment gap [15,16]. These health-system constraints make early identification of MHDs within existing PHC and HIV service platforms especially important.

Evidence from sub-Saharan Africa (SSA) suggests that AYPLHIV experience a substantial burden of common MHDs, although prevalence estimates vary by setting, age, screening tool and cut-off used [17–29]. Zambian studies have reported depressive symptoms and other mental health concerns among young people and people living with HIV, but there remains limited evidence from routine PHC-based adolescent HIV clinics on the prevalence of common MHDs and associated psychosocial, behavioural and HIV-related factors among AYPLHIV [12,14,30,31].

AYPLHIV may experience mental health vulnerability through overlapping biological, social and psychological pathways, including the direct and indirect effects of HIV, the demands of living with a chronic condition, stigma, disclosure concerns, violence, social isolation and treatment fatigue [7,8,17,32–34]. In the Zambian PHC context, opportunistic screening for MHDs among AYPLHIV is not routinely implemented in many HIV care settings, reflecting competing clinical demands, limited mental health staffing and the lack of locally integrated screening and referral models [15,16]. Strategies are therefore needed to support feasible, acceptable and context-appropriate screening within routine HIV services.

Digital platforms may help strengthen screening, referral, monitoring and task-sharing in adolescent health services, including mental health and HIV care [35–38]. However, digital approaches should be implemented within local service-delivery contexts and should not substitute for clear clinical pathways, trained personnel and referral systems.

The AdoMinds study aimed to provide access to MH services and appropriate care by screening for common MHDs (depression, anxiety, post-traumatic stress disorder and substance use) among AYPLHIV and piloting a contextually relevant digital health application, to be used in PHC settings in Zambia. This manuscript focuses on the cross-sectional survey component of AdoMinds; findings from the digital tool co-design, pilot and process evaluation will be reported elsewhere. In this paper, we report the prevalence of common MHDs and associated risk factors among AYPLHIV screened during the cross-sectional survey in Zambia.

## 2. Methods

### 2.1. Ethics statement

Ethical approval was obtained from the University of Zambia Biomedical Research Ethics Committee (UNZABREC) [REF. 3818–2023] and the WHO Ethics Review Committee. Permission to conduct the study was obtained from the Zambian Ministry of Health, including the clinic, district and provincial health offices. Eligible AYPLHIV aged 18 years and older provided written informed consent. Participants younger than 18 years provided written assent, and their parents or guardians provided written consent. Participants who screened positive for severe MHDs or other urgent mental health concerns were referred for clinical assessment and management through the clinic and partner referral pathways described below.

### 2.2. Design

The AdoMinds study was conducted over 14 months from March 2023 to May 2024 using a mixed-methods implementation science approach. The broader study had four phases namely formative work and a cross-sectional survey; co-design and piloting of a digital mental health screening tool; process evaluation; and knowledge translation. This manuscript reports only the cross-sectional survey which aimed to determine the prevalence of common MHDs and examined associated factors among AYPLHIV. The co-design, digital tool pilot and process evaluation components will be reported elsewhere. This cross-sectional study is reported in accordance with the Strengthening the Reporting of Observational Studies in Epidemiology (STROBE) statement [39].

### 2.3. Setting

The study was conducted at Chipata Level 1 Hospital in Chipata compound, a peri-urban community in Lusaka, Zambia. The community has a population of approximately 128,000 people. The hospital runs an HIV clinic that provides comprehensive adult and paediatric services. It is not a referral centre; rather, community members routinely access HIV care, including testing and treatment, as part of their primary healthcare services. At the start of the study in September 2023, there were 583 AYP (69.6% female) aged between 15 and 24 years registered in the clinic’s ART register, receiving treatment. The HIV clinic is served by approximately 15 health care workers (HCWs) namely 9 nurses, 3 clinical officers, 1 doctor and 2 pharmacy technologists. At the beginning of the survey none of the HCWs had been trained to provide MH services.

### 2.4. Study population

AYPLHIV aged 15–24 years enrolled in ART care at Chipata Level 1 Hospital were eligible to participate. We excluded AYP who had a known severe MHD and those who reported being too unwell to answer the questionnaire. We created an enumeration register of all AYPLHIV aged 15–24 years in the national HIV electronic health system (SmartCare Plus), and checked it against paper ART clinic registers to define the eligible population. Participant identification, approach and enrolment are summarised in Fig 1.

**Fig 1:**
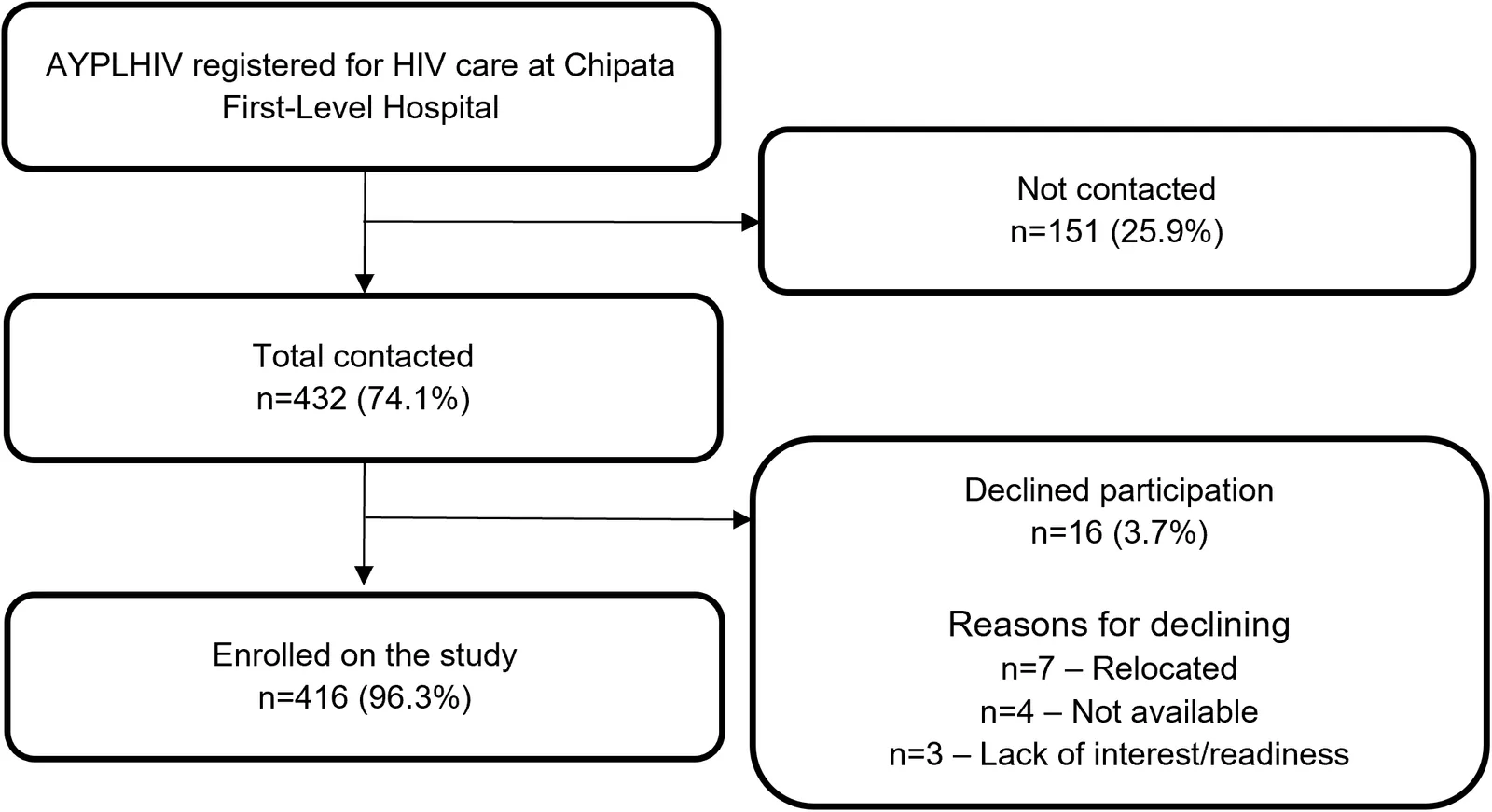
Participant flowchart.

### 2.5. Data collection

#### 2.5.1. Cross-sectional survey.

One trained research assistant administered a tablet-based questionnaire to collect sociodemographic, HIV-treatment, stigma-related, sexual-risk-related and MHD screening data from eligible AYP. The questionnaire was administered in a secluded room at the clinic when adolescents came for scheduled medication collection appointments or when they were invited to the clinic by telephone using contact information obtained from clinic registers. Participant recruitment was conducted from 26 September 2023 to 2 May 2024. The analysis in this manuscript is based on the cross-sectional survey data described here.

#### 2.5.2. MH Screening tools.

We used validated mental health screening tools to identify symptoms of common MHDs. Depression was assessed using the Patient Health Questionnaire-9 (PHQ-9), with a positive screen defined as a score ≥5 to prioritise sensitivity to mild depressive symptoms in this high-risk clinical population. For descriptive severity analyses, PHQ-9 scores were categorised as none/minimal (0–4), mild (5–9), moderate (10–14), moderately severe (15–19) and severe (20–27) [40–43]. Anxiety was assessed using the Generalised Anxiety Disorder 7-item scale (GAD-7), with a positive screen defined as ≥8, consistent with guidance supporting this cut-off for probable anxiety while maintaining sensitivity. GAD-7 severity was categorised as minimal (0–4), mild (5–9), moderate (10–14) and severe (15–21) [44–46]. PTSD was screened using the PTSD Checklist for DSM-5 (PCL-5), with scores >30 classified as above the clinical threshold [47]. Alcohol use was assessed using the Alcohol Use Disorders Identification Test (AUDIT), with scores >7 classified as a positive screen; descriptive categories were 0 (abstainers), 1–7 (low-risk), 8–14 (harmful use) and ≥15 (moderate to severe alcohol use disorder) [48]. Drug use was assessed using the Drug Abuse Screening Test (DAST-10), with scores >0 classified as a positive screen; descriptive categories were 0 (no problems), 1–2 (low level), 3–5 (moderate), 6–8 (substantial) and 9–10 (severe) [48]. The Ask Suicide-Screening Questions (ASQ) Toolkit was used to assess urgent suicide-related risk and trigger referral where indicated [49].

### 2.6. Linkage to care

AYPLHIV who screened positive for severe MHDs for the first time, as defined by the severity cut-off points of the validated MHD screening tools, were referred to a Clinical Officer Psychiatry or Medical Officer based at the clinic. The adolescents were subsequently evaluated and managed at either the clinic or referred to a MH specialist at Chainama psychiatry hospital in Lusaka. Weekly and monthly follow-ups were made for review for AYPLHIV with MHDs. AYPLHIV and their ca re givers were also offered toll-free lines for therapy sessions from local partnering organisations including Lifeline Childline Zambia and Serenity Harm Reduction Program Zambia (SHARPZ).

### 2.7. Quantitative analysis

Data analysis was performed using Stata 18 MP (StataCorp, College Station, TX, USA). Descriptive statistics were used to characterise participants and were presented as frequencies and percentages for categorical variables and as medians with interquartile ranges for continuous variables. Bivariate relationships between MHD outcomes and candidate explanatory variables were examined using chi-square or Fisher exact tests for categorical variables and two-sample t-tests or rank-sum tests for continuous variables, as appropriate. Candidate variables included sociodemographic characteristics (sex, age group, education, marital status, employment, living arrangement and parental loss), HIV-related characteristics (ART duration, ART medication in the previous month and living with another household member with HIV), psychosocial variables (felt stigma, stigmatising attitudes and having someone to talk to), and behavioural or sexual-history variables (ever had sex, forced last sexual encounter and smoking history). A 5% significance level was used for hypothesis tests.

Logistic regression models were used to examine factors associated with having at least one MHD, depression and anxiety. Full adjusted models included all pre-specified candidate variables to allow comparison across exposures. Parsimonious best-fit models were then developed using investigator-led forward and backward selection informed by statistical significance, plausibility and model fit. Variables were considered for entry where they were selected a priori or had p < 0.20 in bivariate analyses, and were retained where they improved model fit based on the Akaike Information Criterion (AIC), showed evidence of association, or were judged important potential confounders. Model fit was assessed using the Hosmer-Lemeshow goodness-of-fit test, and variance inflation factors were examined to assess multicollinearity. Regression models were not fitted for PTSD or substance use because the number of positive screens was small, resulting in inadequate events per variable.

## 3. Results

### 3.1. Quantitative results

#### 3.1.1. Enumeration and consent.

Approximately 583 AYPLHIV aged 15–24 years were registered for HIV care at Chipata Level 1 Hospital, 69.6% of whom were female, with a median age of 21 years (IQR: 18–23). We approached 432 (74.1%) AYP and enrolled 416, while 16 (3.7%) declined to participate. The remaining 151 individuals could not be reached because their contact details were incorrect (addresses and phone numbers) (Fig 1).

#### 3.1.2. Descriptive statistics of study participants.

Among the 416 participants, 268 (64.4%) were female and the median age was 20 years (IQR: 18–23), closely comparable to the distributions among the 583 AYPLHIV registered for care. Of note, 353 (84.9%) AYPLHIV had lost at least one parent, 269 (64.7%) had been on ART for 5 or more years, and about half (49.5%) lived with at least one other household member living with HIV. Additionally, 86 (20.7%) AYPLHIV felt stigmatised because of HIV and 223 (53.6%) had stigmatising attitudes towards PLHIV. Furthermore, 229 (55.0%) AYPLHIV had ever had sex and, among them, 22/229 (9.6%) reported that their last sexual encounter was forced (Table 1).

**Table 1:** Characteristics of study participants.

Characteristic	Frequency N (416)	Percentage (%)
**Sex**		
Male	148	35.6
Female	268	64.4
**Age**	**20**	**(18.0 - 23.0)**
**Age group**		
15 to 17 years	98	23.6
18 to 20 years	137	32.9
21 to 24 years	181	43.5
**Participant lives with**		
Alone	27	6.5
Other relatives	113	27.2
Parents	227	54.6
Husband/wife/boyfriend/girlfriend	45	10.8
Other people	3	0.7
Missing	1	0.2
**Education level**		
None	1	0.2
Primary school	85	20.4
Secondary school	324	77.9
Tertiary	6	1.4
**Marital status**		
Never married/single	360	86.5
In a relationship/single	10	2.4
Married/cohabiting	46	11.1
**Employment status**		
Formally employed	137	32.9
Self-employed	24	5.8
Student/pupil	180	43.3
Unemployed	75	18
**Parent loss**		
Both parents alive	63	15.1
One parent is alive	173	41.6
Both parents are dead	180	43.3
**At least one other household member living with HIV**		
No	207	49.8
Yes	206	49.5
Missing	3	0.7
**ART duration**		
<2 years	63	15.1
2-5 years	74	17.8
5-10 years	103	24.8
>10 years	166	39.9
Missing	10	2.4
**ART medication in previous month**		
No	30	7.2
Yes	385	92.5
Missing	1	0.2
**Feel stigmatised against because of HIV**		
No	330	79.3
Yes	86	20.7
**Has stigmatising attitude towards PLHIV**		
No	193	46.4
Yes	223	53.6
**Had someone to talk to when needed**		
No	135	32.5
Yes	281	67.5
**Ever had sex**		
No	187	45
Yes	229	55
**Last sexual encounter was forced**		
No	207	49.8
Yes	22	5.3
Never had sex	187	45
**Smoking history**		
No	396	95.2
Yes	20	4.8

#### 3.1.3. Prevalence of common MHDs.

Among the AYPLHIV, 297/416 (71.4%) had at least one MHD (Table 2). Fig 2A shows the distribution of the number of positive disorder screens per participant, and 150/416 (36.0%) had two or more disorders simultaneously. A high proportion of participants with at least one common MHD was observed among those who had both parents alive (53/63 [84.1%]), felt stigmatised because of HIV (70/86 [81.4%]), reported that their last sexual encounter was forced (19/22 [86.4%]) and had a smoking history (18/20 [90.0%]) (Table 2).

**Table 2:** Bivariate analysis of common mental health disorders against socio-demographic characteristics of study participants.

Characteristic	Number of participants	≥ 1 Mental Health Disorder n (%)			Depression n (%)			Anxiety n (%)			Post-traumatic Stress Disorder		
		-ve screen	+ve screen	p-value	-ve screen	+ve screen	p-value	-ve screen	+ve screen	p-value	-ve screen	+ve screen	p-value
Overall	416	119 (28.6)	297 (71.4)		137 (32.9)	279 (67.1)		269 (64.7)	147 (35.3)		380 (91.3)	36 (8.7)	
**Sex**				**0.55 C**			**0.78 C**			**0.56 C**			0.004 C*
Male	148	45 (30.4)	103 (69.6)		50 (33.8)	98 (66.2)		93 (62.8)	55 (37.2)		143 (96.6)	5 (3.4)	
Female	268	74 (27.6)	194 (72.4)		87 (32.5)	181 (67.5)		176 (65.7)	92 (34.3)		237 (88.4)	31 (11.6)	
**Age**	**20 (18-23)**	**20 (16-22)**	**20 (18-23)**	**0.24 R**	**20 (16-22)**	**20 (18-23)**	**0.23 R**	**20 (17-23)**	**20 (18-23)**	**0.23 R**	**20 (18-23)**	**19.5 (16-22)**	**0.39 R**
**Age group**				**0.061 C**			0.051 C*			0.029 C*			**0.78 C**
15 to 17 years	98	37 (37.8)	61 (62.2)		42 (42.9)	56 (57.1)		73 (74.5)	25 (25.5)		88 (89.8)	10 (10.2)	
18 to 20 years	137	33 (24.1)	104 (75.9)		39 (28.5)	98 (71.5)		79 (57.7)	58 (42.3)		125 (91.2)	12 (8.8)	
21 to 24 years	181	49 (27.1)	132 (72.9)		56 (30.9)	125 (69.1)		117 (64.6)	64 (35.4)		167 (92.3)	14 (7.7)	
**Participant lives with**				**0.18 C**			**0.27 C**			**0.98 C**			**0.93 E**
Alone	27	7 (25.9)	20 (74.1)		7 (25.9)	20 (74.1)		17 (63.0)	10 (37.0)		25 (92.6)	2 (7.4)	
Other relatives	113	26 (23.0)	87 (77.0)		31 (27.4)	82 (72.6)		74 (65.5)	39 (34.5)		102 (90.3)	11 (9.7)	
Parents	227	75 (33.0)	152 (67.0)		84 (37.0)	143 (63.0)		145 (63.9)	82 (36.1)		207 (91.2)	20 (8.8)	
Husband/wife/boyfriend/girlfriend	45	10 (22.2)	35 (77.8)		14 (31.1)	31 (68.9)		30 (66.7)	15 (33.3)		42 (93.3)	3 (6.7)	
**Education level**				**0.38 E**			**0.64 E**			**0.12 C**			**0.42 E**
Primary school	85	27 (31.8)	58 (68.2)		29 (34.1)	56 (65.9)		62 (72.9)	23 (27.1)		80 (94.1)	5 (5.9)	
Secondary school	324	89 (27.5)	235 (72.5)		105 (32.4)	219 (67.6)		202 (62.3)	122 (37.7)		293 (90.4)	31 (9.6)	
Tertiary	6	3 (50.0)	3 (50.0)		3 (50.0)	3 (50.0)		5 (83.3)	1 (16.7)		6 (100)	0	
**Marital status**				**0.55 E**			**0.91 C**			**0.93 C**			**0.51 E**
Never married/single	360	106 (29.4)	254 (70.6)		120 (33.3)	240 (66.7)		232 (64.4)	128 (35.6)		327 (90.8)	33 (9.2)	
In a relationship/single	10	3 (30.0)	7 (70.0)		3 (30.0)	7 (70.0)		7 (70.0)	3 (30.0)		10 (100)	0	
Married/cohabiting	46	10 (21.7)	36 (78.3)		14 (30.4)	32 (69.6)		30 (65.2)	16 (34.8)		43 (93.5)	3 (6.5)	
**Employment status**				**0.29 C**			**0.12 C**			**0.20 C**			**0.15 E**
Formally employed	137	35 (25.5)	102 (74.5)		41 (29.9)	96 (70.1)		83 (60.6)	54 (39.4)		122 (89.1)	15 (10.9)	
Self-employed	24	8 (33.3)	16 (66.7)		9 (37.5)	15 (62.5)		19 (79.2)	5 (20.8)		23 (95.8)	1 (4.2)	
Student/pupil	180	59 (32.8)	121 (67.2)		69 (38.3)	111 (61.7)		122 (67.8)	58 (32.2)		162 (90.0)	18 (10.0)	
Unemployed	75	17 (22.7)	58 (77.3)		18 (24.0)	57 (76.0)		45 (60.0)	30 (40.0)		73 (97.3)	2 (2.7)	
**Parent loss**				0.038 C*			**0.14 C**			0.049 C*			0.040 C*
Both parents alive	63	10 (15.9)	53 (84.1)		14 (22.2)	49 (77.8)		34 (54.0)	29 (46.0)		53 (84.1)	10 (15.9)	
One parent is alive	173	50 (28.9)	123 (71.1)		59 (34.1)	114 (65.9)		122 (70.5)	51 (29.5)		157 (90.8)	16 (9.2)	
Both parents are dead	180	59 (32.8)	121 (67.2)		64 (35.6)	116 (64.4)		113 (62.8)	67 (37.2)		170 (94.4)	10 (5.6)	
**Registered for HIV care**													
No	0	0	0		0	0		0	0		0	0	
Yes	416	119 (28.6)	297 (71.4)		137 (32.9)	279 (67.1)		269 (64.7)	147 (35.3)		380 (91.3)	36 (8.7)	
**Someone else living with HIV at home**				0.046 C*			0.019 C*			**0.37 C**			**0.72 C**
No	207	50 (24.2)	157 (75.8)		57 (27.5)	150 (72.5)		130 (62.8)	77 (37.2)		190 (91.8)	17 (8.2)	
Yes	206	68 (33.0)	138 (67.0)		79 (38.3)	127 (61.7)		138 (67.0)	68 (33.0)		187 (90.8)	19 (9.2)	
**ART duration**				**0.52 C**			**0.70 C**			**0.53 C**			**0.61 C**
<2 years	63	15 (23.8)	48 (76.2)		17 (27.0)	46 (73.0)		40 (63.5)	23 (36.5)		59 (93.7)	4 (6.3)	
2-5 years	74	25 (33.8)	49 (66.2)		26 (35.1)	48 (64.9)		50 (67.6)	24 (32.4)		66 (89.2)	8 (10.8)	
5-10 years	103	27 (26.2)	76 (73.8)		36 (35.0)	67 (65.0)		61 (59.2)	42 (40.8)		92 (89.3)	11 (10.7)	
>10 years	166	51 (30.7)	115 (69.3)		57 (34.3)	109 (65.7)		112 (67.5)	54 (32.5)		154 (92.8)	12 (7.2)	
**ART medication in the previous month**				**0.82 C**			**0.95 C**			**0.18 C**			**0.35 C**
No	30	8 (26.7)	22 (73.3)		10 (33.3)	20 (66.7)		16 (53.3)	14 (46.7)		26 (86.7)	4 (13.3)	
Yes	385	110 (28.6)	275 (71.4)		126 (32.7)	259 (67.3)		252 (65.5)	133 (34.5)		353 (91.7)	32 (8.3)	
**Feel stigmatised because of HIV**				0.021 C*			0.016 C*			**0.029 C**			**0.050 C**
No	330	103 (31.2)	227 (68.8)		118 (35.8)	212 (64.2)		222 (67.3)	108 (32.7)		306 (92.7)	24 (7.3)	
Yes	86	16 (18.6)	70 (81.4)		19 (22.1)	67 (77.9)		47 (54.7)	39 (45.3)		74 (86.0)	12 (14.0)	
**Has stigmatising attitudes towards PLHIV**				**0.63 C**			**0.46 C**			**0.39 C**			**0.65 C**
No	193	53 (27.5)	140 (72.5)		60 (31.1)	133 (68.9)		129 (66.8)	64 (33.2)		175 (90.7)	18 (9.3)	
Yes	223	66 (29.6)	157 (70.4)		77 (34.5)	146 (65.5)		140 (62.8)	83 (37.2)		205 (91.9)	18 (8.1)	
**Had someone to talk to when needed**				**0.89 C**			**0.58 C**			**0.42 C**			**0.22 C**
No	135	38 (28.1)	97 (71.9)		42 (31.1)	93 (68.9)		91 (67.4)	44 (32.6)		120 (88.9)	15 (11.1)	
Yes	281	81 (28.8)	200 (71.2)		95 (33.8)	186 (66.2)		178 (63.3)	103 (36.7)		260 (92.5)	21 (7.5)	
**Ever had sex**				0.038 C*			0.009 C*			**0.82 C**			**0.95 C**
No	187	63 (33.7)	124 (66.3)		74 (39.6)	113 (60.4)		122 (65.2)	65 (34.8)		171 (91.4)	16 (8.6)	
Yes	229	56 (24.5)	173 (75.5)		63 (27.5)	166 (72.5)		147 (64.2)	82 (35.8)		209 (91.3)	20 (8.7)	
**Last sexual encounter was forced**				**0.058 C**			0.012 C*			**0.59 C**			**1.00 C**
No	207	53 (25.6)	154 (74.4)		60 (29.0)	147 (71.0)		135 (65.2)	72 (34.8)		189 (91.3)	18 (8.7)	
Yes	22	3 (13.6)	19 (86.4)		3 (13.6)	19 (86.4)		12 (54.5)	10 (45.5)		20 (90.9)	2 (9.1)	
Never had sex	187	63 (33.7)	124 (66.3)		74 (39.6)	113 (60.4)		122 (65.2)	65 (34.8)		171 (91.4)	16 (8.6)	
**Smoking history**				**0.059 E**			**0.44 C**			**0.97 C**			**0.55 E**
No	396	117 (29.5)	279 (70.5)		132 (33.3)	264 (66.7)		256 (64.6)	140 (35.4)		361 (91.2)	35 (8.8)	
Yes	20	2 (10.0)	18 (90.0)		5 (25.0)	15 (75.0)		13 (65.0)	7 (35.0)		19 (95.0)	1 (5.0)	

P-values were calculated using Pearson’s chi-squared test (C), Fisher’s exact test (E), or Wilcoxon rank-sum test (R), as appropriate. An asterisk (*) denotes statistical significance at p < 0.05. Percentages are row percentages unless otherwise specified.

**Fig 2:**
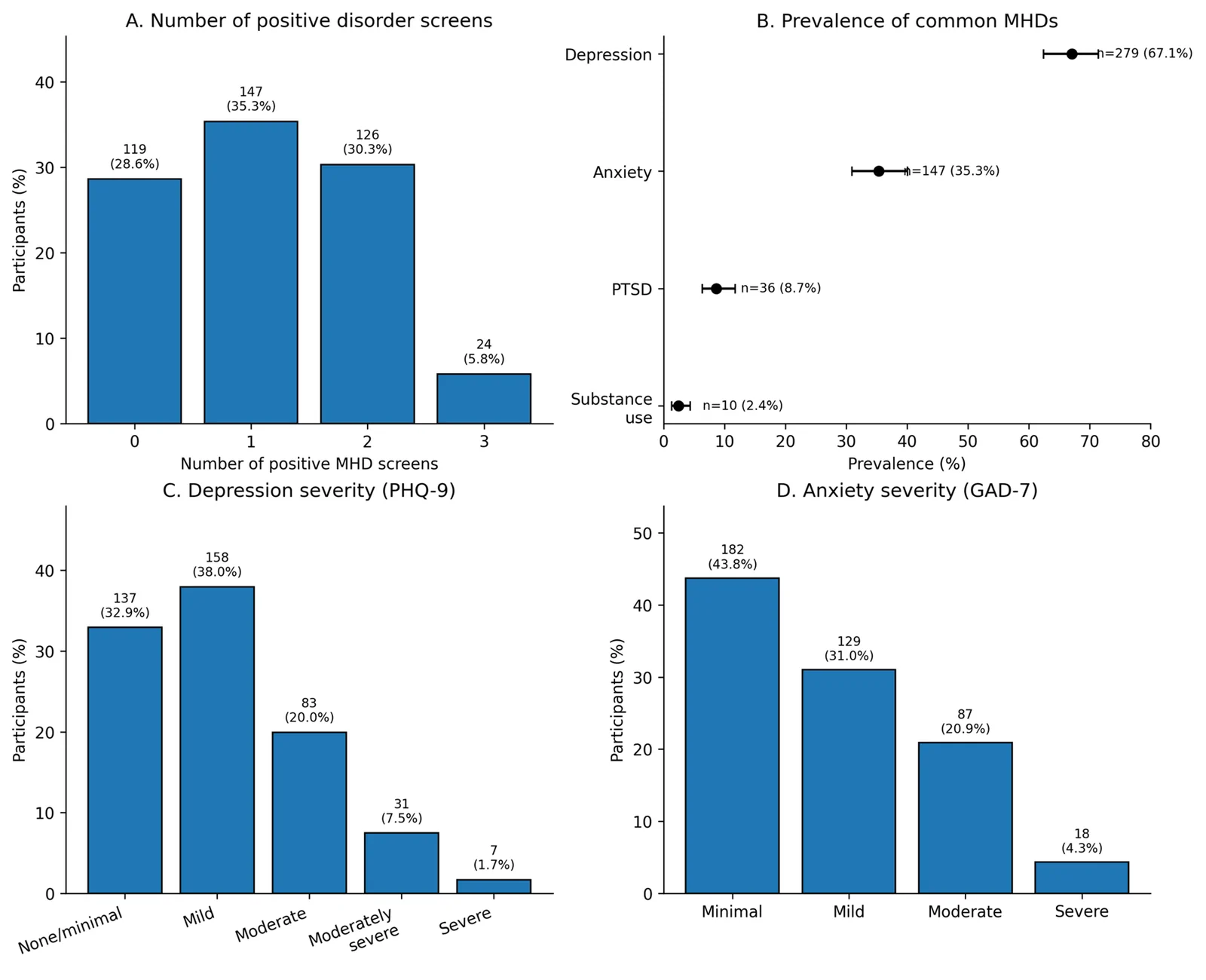
Prevalence and severity of common mental health disorders among AYPLHIV. Panel A shows the number of positive disorder screens per participant. Substance use denotes a positive screen on AUDIT (>7) and/or DAST-10 (>0). Panel B shows the prevalence of depression, anxiety, PTSD and substance use with 95% confidence intervals. Panel C shows depression severity based on PHQ-9 categories (0-4 none/minimal, 5-9 mild, 10-14 moderate, 15-19 moderately severe and 20-27 severe). Panel D shows anxiety severity based on GAD-7 categories (0-4 minimal, 5-9 mild, 10-14 moderate and 15-21 severe).

Depression affected 279 (67.1% [95% CI: 62.1%, 71.6%]) AYPLHIV and was the most prevalent disorder (Fig 2B). The proportion of AYPLHIV experiencing depression was higher among those who lived alone (20/27 [74.1%]), those who had both parents alive (49/63 [77.8%]), were unemployed (57/75 [76.0%]), felt stigmatised because of HIV (67/86 [77.9%]), were forced into their last sexual encounter (19/22 [86.4%]), and were smokers (15/20 [75.0%]) (Table 2). As many as 121/416 (29.2%) AYPLHIV had moderate depression or worse, with 31/416 (7.5%) experiencing moderately severe depression and 7/416 (1.7%) experiencing severe depression (Fig 2C).

The second most prevalent MHD was anxiety, which affected 147 (35.3% [95% CI: 30.7%, 40.1%]) AYPLHIV (Fig 2B). The proportion of AYPLHIV experiencing anxiety was higher among those aged 18–20 years (58/137 [42.3%]), those who felt stigmatised because of HIV (39/86 [45.3%]) and those who had not been taking ART in the previous month (14/30 [46.7%]) compared with those who were taking medication (Table 2). A quarter of AYPLHIV (105/416 [25.2%]) experienced moderate anxiety or worse, with 87/416 (20.9%) experiencing moderate anxiety and 18/416 (4.3%) experiencing severe anxiety (Fig 2D).

The third most prevalent disorder was PTSD, affecting 36 (8.7% [95% CI: 6.1%, 11.8%]) AYPLHIV (Fig 2B). Higher proportions of PTSD were observed among those who felt stigmatised because of HIV (12/86 [14.0%]), those who still had both parents alive (10/63 [15.9%]) and those without anyone to talk to (15/135 [11.1%]) compared with their peers in the opposite groups (Table 2). Substance use was the least prevalent disorder, with only 10 (2.4% [95% CI: 1.2%, 4.4%]) participants screening positive (Fig 2B).

#### 3.1.4. Factors associated with common MHDs.

HIV-related stigma was consistently associated with MHD outcomes. AYPLHIV who felt stigmatised because of their HIV status had higher odds of having at least one MHD (AOR = 1.99, 95% CI: 1.10-3.63; p = 0.024) (Table 3). Felt stigma was also associated with depression (AOR = 1.77, 95% CI: 1.01-3.13; p = 0.048) and anxiety (AOR = 1.73, 95% CI: 1.05-2.85; p = 0.032) (Table 4). Living with at least one other household member with HIV was associated with lower odds of depression (AOR = 0.64, 95% CI: 0.42-0.97; p = 0.036) (Table 4).

**Table 3:** Factors associated with having at least 1 disorder among adolescents living with HIV.

Characteristic	≥ 1 Mental health disorder					
	Unadjusted		Adjusted full model^*†*^		Adjusted best-fit model^*‡*^	
	OR (95% CI)	p-value	OR (95% CI)	p-value	OR (95% CI)	p-value
**Sex**						
Male	Ref	–	Ref	‘-		
Female	1.15 (0.74, 1.78)	0.546	1.17 (0.68, 2.01)	0.565		
**Age group**						
15 to 17 years	Ref	–	Ref	‘-		
18 to 20 years	1.91 (1.09, 3.37)	0.025*	1.59 (0.75, 3.37)	0.227		
21 to 24 years	1.63 (0.97, 2.76)	0.066	1.12 (0.45, 2.77)	0.811		
**Participant lives with**						
Alone	Ref	–	Ref	‘-		
Other relatives	1.17 (0.45, 3.08)	0.748	1.20 (0.37, 3.84)	0.761		
Parents	0.71 (0.29, 1.75)	0.457	1.06 (0.35, 3.21)	0.922		
Husband/wife/boyfriend/girlfriend	1.23 (0.40, 3.72)	0.720’	1.55 (0.43, 5.50)	0.500		
**Education level**						
Primary school	Ref	–	Ref	‘-		
Secondary school	1.23 (0.73, 2.06)	0.435	1.33 (0.74, 2.40)	0.336		
**Marital status**						
Never married/single	Ref	–	Ref	‘-		
In a relations/single	0.97 (0.25, 3.84)	0.970’	1.70 (0.74, 2.40)	0.336		
Married/cohabiting	1.50 (0.72, 3.14)	0.279	‘-	‘-		
**Employment status**						
Formally employed	Ref	–	Ref	‘-		
Self-employed	0.69 (0.27, 1.74)	0.428	0.69 (0.27, 1.78)	0.440		
Student/pupil	0.70 (0.43, 1.15)	0.163	1.52 (0.73, 3.16)	0.267		
Unemployed	1.17 (0.60, 2.27)	0.641	1.66 (0.72, 3.85)	0.238		
**Parent loss**						
Both parents alive	Ref	–	Ref	–	Ref	–
One parent is alive	0.46 (0.22, 0.98)	0.045	0.46 (0.18, 1.13)	0.092	0.46 (0.21, 0.97)	0.042*
Both parents are dead	0.39 (0.18, 0.81)	0.012*	0.40 (0.16, 1.02)	0.054	0.38 (0.18, 0.80)	0.010*
**Someone else living with HIV at home**						
No	Ref	–	Ref	–		
Yes	0.65 (0.42, 0.99)	0.047*	0.75 (0.44, 1.27)	0.284		
**ART duration**						
<2 years	Ref	–	Ref	–		
2-5 years	0.61 (0.29, 1.30)	0.202	0.60 (0.26, 1.37)	0.225		
5-10 years	0.88 (0.43, 1.82)	0.730’	0.99 (0.44, 2.23)	0.990		
>10 years	0.70 (0.36, 1.37)	0.304	0.79 (0.37, 1.69)	0.545		
**ART medication in previous month**						
No	Ref	–	Ref	–		
Yes	0.91 (0.39, 2.10)	0.824	0.86 (0.38, 1.94)	0.715		
**Feel stigmatised against because of HIV**						
No	Ref	–	Ref	–	Ref	–
Yes	1.99 (1.10, 3.58)	0.023*	2.03 (0.38, 1.94)	0.042*	1.99 (1.10, 3.63)	0.024*
**Has stigmatising attitude towards PLHIV**						
No	Ref	–	Ref	–		
Yes	0.90 (0.59, 1.38)	0.631	0.79 (0.48, 1.32)	0.475		
**Ever had sex**						
No	Ref	–	Ref	–		
Yes	1.57 (1.02, 2.41)	0.039*	1.27 (0.66, 2.46)	0.470		
**Last sexual encounter was forced**						
No	Ref	–	Ref	–		
Yes	2.18 (0.62, 7.66)	0.224	3.21 (0.72, 14.46)	0.129		
Never had sex	0.68 (0.44, 1.05)	0.079	–	–		
**Smoking history**						
No	Ref	–	Ref	–	Ref	–
Yes	3.77 (0.86, 16.53)	(0.078)	4.58 (0.99, 21.21)	0.052	4.24 (0.98, 18.30)	0.053

Note: OR odds ratio, CI confidence interval, Ref reference, ART antiretroviral therapy, PLHIV people living with HIV. *p < 0.05. Full adjusted models included all measured variables; best-fit models retained variables selected using investigator-led selection informed by p-values, plausibility and AIC.

**Table 4:** (a) Factors associated with depression among adolescents and young people living with HIV.

Characteristic	Unadjusted OR (95% CI)	p-value	Adjusted full OR (95% CI)	p-value	Adjusted best-fit OR (95% CI)	p-value
**a**						
**Sex**						
Male	Ref	-	Ref	-		
Female	1.06 (0.69, 1.63)	0.784	0.96 (0.57, 1.63)	0.982		
**Age group**						
15 to 17 years	Ref	-	Ref	-		
18 to 20 years	1.88 (1.09, 3.25)	0.023*	1.39 (0.69, 2.81)	0.362		
21 to 24 years	1.66 (0.99, 2.76)	0.051*	0.97 (0.41, 2.28)	0.935		
**Participant lives with**						
Alone	Ref	-	Ref	-		
Other relatives	0.93 (0.36, 2.41)	0.874	1.13 (0.36, 3.57)	0.829		
Parents	0.60 (0.24, 1.47)	0.26	1.04 (0.35, 3.13)	0.940		
Spouse/partner	0.78 (0.27, 2.25)	0.64	1.01 (0.29, 3.50)	0.987		
**Education level**						
Primary school	Ref	-	Ref	-		
Secondary school	1.08 (0.65, 1.79)	0.765	1.14 (0.65, 2.01)	0.640		
**Marital status**						
Never married/single	Ref	-	Ref	-		
In a relations/single	1.17 (0.30, 4.61)	0.821	2.14 (0.29, 15.74)	0.455		
Married/cohabiting	1.15 (0.59, 2.23)	0.685	-	-		
**Employment status**						
Formally employed	Ref	-	Ref	-		
Self-employed	0.71 (0.29, 1.76)	0.461	0.72 (0.27, 1.88)	0.500		
Student/pupil	0.69 (0.43, 1.10)	0.12	1.25 (0.63, 2.49)	0.517		
Unemployed	1.35 (0.71, 2.57)	0.358	1.73 (0.80, 3.74)	0.167		
**Parent loss**						
Both parents alive	Ref	-	Ref	-		
One parent is alive	0.55 (0.28, 1.08)	0.083	0.55 (0.25, 1.22)	0.144		
Both parents are dead	0.51 (0.26, 1.00)	0.050*	0.54 (0.24, 1.23)	0.141		
Other household member living with HIV						
No	Ref	-	Ref	-	Ref	-
Yes	0.61 (0.40, 0.92)	0.020*	0.72 (0.44, 1.19)	0.203	0.64 (0.42, 0.97)	0.036*
**ART duration**						
<2 years	Ref	-	Ref	-		
2-5 years	0.68 (0.33, 1.42)	0.307	0.65 (0.29, 1.46)	0.298		
5-10 years	0.69 (0.35, 1.37)	0.286	0.72 (0.34, 1.54)	0.401		
>10 years	0.71 (0.37, 1.34)	0.289	0.76 (0.36, 1.58)	0.462		
**ART medication in previous month**						
No	Ref	-	Ref	-		
Yes	1.02 (0.47, 2.25)	0.953	0.99 (0.42, 2.35)	0.994		
**Feel stigmatised against because of HIV**						
No	Ref	-	Ref	-	Ref	-
Yes	1.97 (1.13, 3.44)	0.017*	1.89 (1.02, 3.51)	0.044*	1.77 (1.01, 3.13)	0.048*
**Has stigmatising attitude towards PLHIV**						
No	Ref	-	Ref	-		
Yes	0.86 (0.57, 1.29)	0.457	0.82 (0.51, 1.32)	0.410		
**Ever had sex**						
No	Ref	-	Ref	-	Ref	-
Yes	1.72 (1.14, 2.59)	0.010*	1.53 (0.82, 2.85)	0.184	1.59 (1.04, 2.42)	0.032*
**Last sexual encounter was forced**						
No	Ref	-	Ref	-		
Yes	2.60 (0.74, 9.12)	0.135	3.89 (0.92, 16.47)	0.065		
Never had sex	0.63 (0.41, 0.95)	0.027*	-	-		
**Smoking history**						
No	Ref	-	Ref	-		
Yes	1.50 (0.53, 4.22)	0.442	1.42 (0.45, 4.48)	0.546		
**Table (b) Factors associated with anxiety among adolescents and young people living with HIV.**						
**Sex**						
Male	Ref	-	Ref	-		
Female	0.88 (0.58, 1.34)	0.563	0.86 (0.50, 1.49)	0.603		
**Age group**						
15 to 17 years	Ref	-	Ref	-	Ref	-
18 to 20 years	2.14 (1.22, 3.78)	0.008*	2.04 (0.97, 4.30)	0.061	1.83 (1.02, 3.29)	0.042*
21 to 24 years	1.60 (0.92, 2.76)	0.093	1.43 (0.59, 3.45)	0.428	1.32 (0.75, 2.35)	0.337
**Participant lives with**						
Alone	Ref	-	Ref	-		
Other relatives	0.89 (0.37, 2.14)	0.805	0.89 (0.29, 2.72)	0.840		
Parents	0.96 (0.42, 2.20)	0.926	1.73 (0.59, 5.12)	0.319		
Spouse/partner	0.85 (0.31, 2.30)	0.749	1.30 (0.39, 4.35)	0.674		
**Education level**						
Primary school	Ref	-	Ref	-	Ref	-
Secondary school	1.63 (0.96, 2.76)	0.071	2.06 (1.15, 3.72)	0.016*	1.57 (0.91, 2.70)	0.106
**Marital status**						
Never married/single	Ref	-	Ref	-		
In a relations/single	0.78 (0.20, 3.08)	0.726	1.09 (0.20, 6.03)	0.924		
Married/cohabiting	0.97 (0.51, 1.86)	0.937	-	-		
**Employment status**						
Formally employed	Ref	-	Ref	-		
Self-employed	0.41 (0.15, 1.17)	0.096	0.47 (0.16, 1.39)	0.170		
Student/pupil	0.74 (0.47, 1.19)	0.214	0.73 (0.36, 1.45)	0.367		
Unemployed	1.04 (0.59, 1.86)	0.884	1.35 (0.67, 2.71)	0.396		
**Parent loss**						
Both parents alive	Ref	-	Ref	-	Ref	-
One parent is alive	0.49 (0.27, 0.89)	0.019*	0.41 (0.20, 0.83)	0.014*	0.52 (0.28, 0.94)	0.032*
Both parents are dead	0.68 (0.38, 1.22)	0.202	0.56 (0.27, 1.17)	0.122	0.74 (0.41, 1.33)	0.308
Other household member living with HIV						
No	Ref	-	Ref	-		
Yes	0.83 (0.56, 1.25)	0.373	0.83 (0.50, 1.36)	0.456		
**ART duration**						
<2 years	Ref	-	Ref	-		
2-5 years	0.83 (0.41, 1.69)	0.617	0.79 (0.35, 1.79)	0.579		
5-10 years	1.20 (0.63, 2.28)	0.585	1.33 (0.62, 2.84)	0.459		
>10 years	0.84 (0.46, 1.54)	0.57	0.77 (0.36, 1.63)	0.488		
**ART medication in previous month**						
No	Ref	-	Ref	-		
Yes	0.60 (0.28, 1.26)	0.179	0.47 (0.20, 1.09)	0.077		
**Feel stigmatised against because of HIV**						
No	Ref	-	Ref	-	Ref	-
Yes	1.72 (1.06, 2.79)	0.028*	1.96 (1.12, 3.44)	0.019*	1.73 (1.05, 2.85)	0.032*
**Has stigmatising attitude towards PLHIV**						
No	Ref	-	Ref	-		
Yes	1.19 (0.80, 1.79)	0.388	1.31 (0.82, 2.10)	0.258		
**Ever had sex**						
No	Ref	-	Ref	-		
Yes	1.03 (0.69, 1.55)	0.871	0.69 (0.37, 1.29)	0.248		
**Last sexual encounter was forced**						
No	Ref	-	Ref	-		
Yes	1.58 (0.65, 3.85)	0.309	2.25 (0.79, 6.42)	0.131		
Never had sex	1.01 (0.67, 1.54)	0.951				
**Smoking history**						
No	Ref	-	Ref	-		
Yes	0.98 (0.38, 2.52)	0.974	0.88 (0.31, 2.45)	0.803		

OR odds ratio, CI confidence interval, Ref reference. * Statistically significant at 5% significance level. Full adjusted models included all measured variables; best-fit models retained variables selected through investigator-led selection informed by p-values, plausibility and AIC.

Sexual-history variables showed patterns that require cautious interpretation. Ever having had sexual intercourse was associated with higher odds of depression (AOR = 1.59, 95% CI: 1.04-2.42; p = 0.032) (Table 4). Participants who reported that their last sexual encounter was forced had higher odds of having at least one MHD (AOR = 3.21, 95% CI: 0.72-14.46; p = 0.129), anxiety (AOR = 2.25, 95% CI: 0.79-6.42; p = 0.131) and depression (AOR = 3.89, 95% CI: 0.92-16.47; p = 0.065), but these estimates did not meet the conventional 5% threshold for statistical significance.

Some sociodemographic characteristics were associated with common MHD outcomes. Anxiety was more likely among participants aged 18–20 years than among those aged 15–17 years (AOR = 1.83, 95% CI: 1.02-3.29; p = 0.042) (Table 4). Compared with participants with both parents alive, those who had lost one parent (AOR = 0.46, 95% CI: 0.21-0.97; p = 0.042) or both parents (AOR = 0.38, 95% CI: 0.18-0.80; p = 0.010) had lower odds of having at least one MHD (Table 3). A similar pattern was observed for anxiety among those who had lost one parent (AOR = 0.52, 95% CI: 0.28-0.94; p = 0.032) (Table 4). These findings should be interpreted cautiously because of the cross-sectional design and the possibility of unmeasured confounding, including differences in living arrangements, family support and survivorship among clinic-attending AYPLHIV. We did not fit regression models for PTSD because there were few positive screens; however, bivariate analyses showed associations with sex (p = 0.004) and parental loss (p = 0.040) (Table 2).

## 4. Discussion

We sought to determine the prevalence of common MHDs and associated factors among AYPLHIV receiving routine HIV care at PHC level in Lusaka, Zambia. We found a high burden of symptoms of common MHDs, with 71.4% of participants screening positive for at least one MHD. Depression and anxiety were the most prevalent conditions. HIV-related stigma was consistently associated with higher odds of MHD outcomes, while associations involving forced sex, smoking history and parental loss require cautious interpretation because of imprecision, potential residual confounding and the cross-sectional design.

The prevalence of depression among AYPLHIV in this study was 67.3% using the PHQ-9 with a cut-off score of>=5. This cut-off prioritises sensitivity and captures mild depressive symptoms; therefore, comparisons with studies using higher PHQ-9 thresholds or different screening tools should be made cautiously. A survey among 103 AYPLHIV at a hospital in Choma District, Zambia reported a high burden of mental health problems [18]. Other studies among AYPLHIV in South Africa, Tanzania, Kenya and Mozambique have also reported substantial depressive symptoms, although prevalence estimates differ by setting, age distribution, instrument and cut-off used [19,20,24,27–29].

In our study, anxiety was prevalent among 35.3% of AYPLHIV using the GAD-7. This finding aligns closely with a study from Botswana, which reported a 33.1% prevalence of anxiety among AYPLHIV using the GAD-7 tool [23]. Differences between our findings and other studies may reflect variation in clinic context, participant age, HIV disclosure and stigma experiences, screening thresholds and the availability of psychosocial support.

Post-traumatic stress disorder was observed among 8.7% of AYPLHIV in our study. This was consistent with findings from a recent systematic review of MHDs among AYPLHIV which found the prevalence to be 10.5% (95% CI: 5.8–15.2%) [50]. Our prevalence findings of substance use of 2.4% were, however, much lower than other findings [51].

We found that HIV-related stigma was strongly associated with all the MHDs we measured. HIV-related stigma is a known risk factor for both HIV and MHDs [52]. It is associated with depressive symptoms, anxiety and other conditions and has effects on HIV treatment outcomes [17]. While there have been efforts to combat it, it remains high and has significant effects on AYPLHIV [53–56]. Like other African countries, mental disorders remain a silent condition in Zambia and it has been found that the mentally ill suffer in silence due to the negative attitudes attached to mental illness [57]. The mentally ill are scared to disclose a mental illness due to fear of being isolated from society [58]. Innovative methods such as those leveraging collaborative socioecological models for awareness and empathy building [59] should be applied to subdue it at all levels.

The finding that parental loss was associated with lower odds of some MHD outcomes was unexpected and should not be interpreted as evidence that parental loss is protective in general. Studies from other settings have shown that parental loss can increase vulnerability to depression and anxiety [60,61]. In our setting, this pattern may reflect unmeasured differences in living arrangements, caregiving stability, family disclosure dynamics, social support or clinic attendance among AYPLHIV. It is also possible that participants with both parents alive experienced other forms of family stress or HIV-related disclosure challenges that were not captured in the survey. Further qualitative and longitudinal work is needed to understand this finding.

We identified an association between MHDs and sexual behaviours. Most AYP experiencing MHDs tend to exhibit risky sexual behaviour but the temporal link between the phenomena is not well established and appears bidirectional [12,62,63]. Consistent with our findings, studies from different settings have demonstrated that adolescents who are exposed to sexual violence are more likely to experience MHDs like depression, suicidal behaviour, anti-social behaviour and substance use [64,65]. Such MHDs have been shown to persist into adulthood indicating long-term deleterious effects [66]. Evidence-based interventions to reduce sexual violence among this key population need to be considered [67].

The high prevalence of common MHDs in this PHC-based HIV clinic highlights the need for screening approaches that can be integrated into routine adolescent HIV care. Although the broader AdoMinds project included co-design and piloting of a digital mental health screening tool, this manuscript does not evaluate the feasibility or acceptability of that tool. Future reports from the implementation components of AdoMinds will examine whether digital, task-shared screening approaches are feasible and acceptable from the perspectives of AYPLHIV, healthcare workers and mental health experts.

Participant recruitment was affected by incomplete or inaccurate contact information in ART registers, including missing or incorrect telephone numbers and addresses. Some potential participants did not collect medication personally, which reduced opportunities for direct contact. These challenges may have introduced selection bias if AYPLHIV who were unreachable differed systematically from those who were enrolled. To mitigate these recruitment challenges, study staff worked with clinic teams and community advisory structures, used telephone follow-up where contact details were available, and aligned recruitment with ART support group days and clinic visits. These mitigation strategies improved reach but could not fully remove the potential for selection bias.

### 4.1. Strengths and limitations

The main limitations are the cross-sectional design, which prevents causal inference, and reliance on screening tools rather than diagnostic clinical interviews. Some HIV-treatment variables were derived from SmartCare Plus and paper ART registers, which had missing or incomplete entries for some participants. The survey also did not capture the specific relationship of other household members living with HIV, limiting interpretation of this variable. Some exposures, including forced sex and smoking history, had small cell counts, resulting in wide confidence intervals and imprecise estimates.

The study also has important strengths. It was conducted within a routine PHC HIV clinic, enrolled a large proportion of AYPLHIV registered for care at the facility, used validated mental health screening tools and linked participants with severe symptoms or urgent concerns to available referral pathways. These features make the findings relevant to similar adolescent HIV care settings in Zambia and other SSA contexts.

### 4.2. Recommendations

This study supports the need for integrated screening for common MHDs among AYPLHIV and highlights the importance of strengthening referral pathways within routine HIV care. Future research should evaluate scalable models for screening and linkage to care, including task-sharing approaches and digital tools, while assessing acceptability, cost, health system requirements and effects on HIV and mental health outcomes.

## 5. Conclusion

Common MHDs were highly prevalent among AYPLHIV seeking routine HIV care at the PHC level in Lusaka, Zambia, with depression the most prevalent MHD. HIV-related stigma was consistently associated with higher odds of MHD outcomes. There was borderline evidence that smoking and forced sex were associated with higher odds of MHD outcomes, while parental loss was associated with lower odds of having at least one MHD. Integrating mental health screening and referral pathways into adolescent HIV services could support earlier identification and care, but implementation models, including digital and task-shared approaches, require further evaluation.

## Abbreviations

AIC: Akaike Information Criterion
ART: Antiretroviral Therapy
ASQ: Ask Suicide-Screening Questions
AUDIT: Alcohol Use Disorders Identification Test
AYP: Adolescents and Young People
AYPLHIV: Adolescents and Young People Living with HIV
DAST-10: Drug Abuse Screening Test
GAD-7: Generalised Anxiety Disorder-7
GOF: Goodness of Fit
HCWs: Healthcare Workers
HIV: Human Immunodeficiency Virus
LMIC: Low- and Middle-Income Countries
MH: Mental Health
MHDs: Mental Health Disorders
NCD: Non-Communicable Diseases
PCL-5: PTSD Checklist for DSM-5
PHC: Primary Healthcare
PHQ-9: Patient Health Questionnaire-9
PTSD: Post-Traumatic Stress Disorder
SHARPZ: Serenity Harm Reduction Program Zambia
SSA: Sub-Saharan Africa
UNZABREC: University of Zambia Biomedical Research Ethics Committee
WHO: World Health Organization.
